# Risk factors for bone cement displacement after percutaneous vertebral augmentation for osteoporotic vertebral compression fractures

**DOI:** 10.3389/fsurg.2022.947212

**Published:** 2022-07-28

**Authors:** Xiangcheng Gao, Jinpeng Du, Lin Gao, Dingjun Hao, Hua Hui, Baorong He, Liang Yan

**Affiliations:** ^1^ Department of Spine Surgery, Honghui Hospital, Xi’an Jiaotong University, Xi’an, China; ^2^ Medical College, Yan'an University, Yan'an, China

**Keywords:** osteoporotic vertebral compression fracture (OVCF), percutaneous vertebral augmentation, complication, risk factors, bone cement displacement

## Abstract

**Purpose:**

To explore the risk factors of bone cement displacement after percutaneous vertebral augmentation (PVA) in patients with osteoporotic vertebral compression fracture (OVCF).

**Methods:**

We retrospectively reviewed the records of 1,538 patients with OVCF treated with percutaneous vertebroplasty (PVP) or percutaneous vertebroplasty (PKP) from January 2016 to June 2021. Patients were divided into bone cement displacement group (*n *= 78) and bone cement non-displacement group (*n *= 1,460) according to the radiographic images. Possible risk factors for bone cement displacement were noted, including age, gender, body mass index (BMI), bone mineral density (BMD), underlying disease, number of fractured vertebrae, involved vertebral segment, surgical method, surgical approach, vertebral height, Cobb angle, cement leakage, the viscosity of bone cement, bone cement diffuse ratio, degree of bone cement interweaving, sagittal bone cement placement, targeted location of bone cement, the distance between the bone cement and the upper and lower endplates, the time of wearing brace and postoperative osteoporosis treatment. Risk factors were identified with univariate and multivariate logistic regressions and the discrimination ability of the predictive indicators was evaluated using area under the curve (AUC) of the receiver operating characteristic (ROC).

**Results:**

In multivariate regression, independent risk factors for bone cement displacement included: high restoration of Cobb angle (OR = 2.019, 95%[CI] 1.545–4.852, *P* < 0.001), cement leakage (anterior edge) (OR = 1.727, 95%[CI] 1.05–2.20, *P* < 0.001), small degree of bone cement interweaving (OR = 1.917, 95%[CI] 1.129–2.747, *P* < 0.001), non-targeted location of bone cement (OR = 2.323, 95%[CI] 1.645–4.134, *P* < 0.001), short duration of brace wearing (OR = 3.207, 95%[CI] 2.036–4.348, *P* < 0.001) and postoperative osteoporosis treatment (OR = 0.422, 95% CI = 0.323–0.547, *P* < 0.001). The AUCs for the high restoration of Cobb angle, cement leakage (anterior edge), small degree of bone cement interweaving, non-targeted location of bone cement, short duration of brace wearing and non-postoperative osteoporosis treatment were 0.784 (95% CI, 0.747–0.821), 0.811 (95% CI 0.764–0.859), 0.917 (95%CI 0.864–0.970), 0.610 (95%CI 0.552–0.669), 0.854 (95%CI 0.816–0.892) and 0.756 (95% CI, 0.712–0.800), respectively.

**Conclusion:**

High restoration of Cobb angle, cement leakage (anterior edge), small degree of bone cement interweaving, non-targeted location of bone cement, short duration of brace wearing and non-postoperative osteoporosis treatment were the independent risk factors of bone cement displacement after PVA.

## Introduction

Osteoporosis is a common systemic skeletal disorder, characterized by bone fragility and increased fracture risk, and it is the major cause of vertebral compression fractures in older adults (1, 2). Osteoporotic vertebral compression fracture (OVCF) is the most common complication of osteoporosis and account for almost half of osteoporotic fractures annually. Approximately 1,416,000 OVCFs occur yearly around the world, and about 40% of women experience OVCFs in their lifetime (3). Furthermore, with the increasingly serious aging of the population, its incidence is increasing annually that will lead to an increase in healthcare costs (4). It is known to affect quality of life and increase mortality by causing continuous pain, limited ambulation, and progression of kyphosis (5). Hence, proper diagnosis and treatment are required.

Treatment for OVCF includes conservative and surgical approaches. Most patients improve with conservative treatments involving bed rest, immobilization, analgesics, and thoracolumbar bracing while fracture healing occurs. However, some patients with acute symptomatic OVCF and some patients with persistent pain after conservative treatment are managed with surgery to avoid bedridden complications such as pressure ulcers, pneumonia, and deep vein thrombosis. Percutaneous vertebral augmentation (PVA) is a classic minimally invasive procedure to reduce pain and further collapse and/or renew vertebral body height, including percutaneous vertebroplasty (PVP) and percutaneous kyphoplasty (PKP), which involves the percutaneous injection of polymethylmethacrylate (PMMA) into the fractured vertebra through large cannulated needles (6, 7). Although PVA provides quick pain relief and improved physical function, some postoperative complications can still occur including bone cement leakage, bone cement implantation syndrome, infection, thermal damage to surrounding soft tissue, and adjacent vertebral fracture (8).

Although bone cement displacement is a rare complication, some scholars believed that it can cause vertebral collapse, local instability of the spine, and pseudojoint formation, which may lead to intractable pain, aggravation of kyphosis, and even neurological impairment (9, 10). To date, only cases of bone cement displacement after PVA have been reported, which factors are closely related to the occurrence of bone cement displacement has not been determined (9–11). In short, there are no systematic studies on bone cement displacement after PVA, a lack of quantitative definition of bone cement displacement, and clinicians do not pay enough attention to bone cement displacement after PVA. Therefore, based on a large dataset from our hospital, we collected the clinical data of 1,538 patients with OVCF treated in Honghui Hospital affiliated to Xi’an Jiaotong University from January 2016 to June 2021. We analyzed the clinical data of patients in a case-control study to determine the factors associated with bone cement displacement after PVA in patients with OVCF. The purpose of the study is as follow: (i) to analyze the risk factors of bone cement displacement after PVA and (ii) to increase patient and physician awareness of cement displacement, thereby reducing its occurrence by avoiding risk factors.

## Data and methods

### General information

Inclusion criteria: (1) Preoperative low back pain, accompanied by unable to turn over, or weak feeling of standing up local spinous process buckle tenderness. (2) The fractured vertebral body conforms to the imaging characteristics, that is, MRI showed low T1 signal and high T2 signal, and the above symptoms were combined. (3) Bone mineral density (BMD) was measured by dual-energy x-ray absorptiometry with T value ≤−2.5SD, combined with low energy fractures. (4) Treated with PVP or PKP. Exclusion criteria: (1) Pathological fracture. (2) Old fracture. (3) Burst fracture. (4) Incomplete clinical data.

### Diagnostic criteria of bone cement displacement

(1) x-ray film showed rupture of the anterior cortex of vertebral body and anterior displacement of bone cement. (2) CT showed rupture of the prevertebral cortex, with the cement leading edge more than 2 mm from the anterior edge of the vertebral body. (3) MRI showed vertebral collapse, sagittal T1-weighted image and T2-weighted image of the fracture cavity showed abnormally low signal and high signal, respectively.

**Figure 1:**
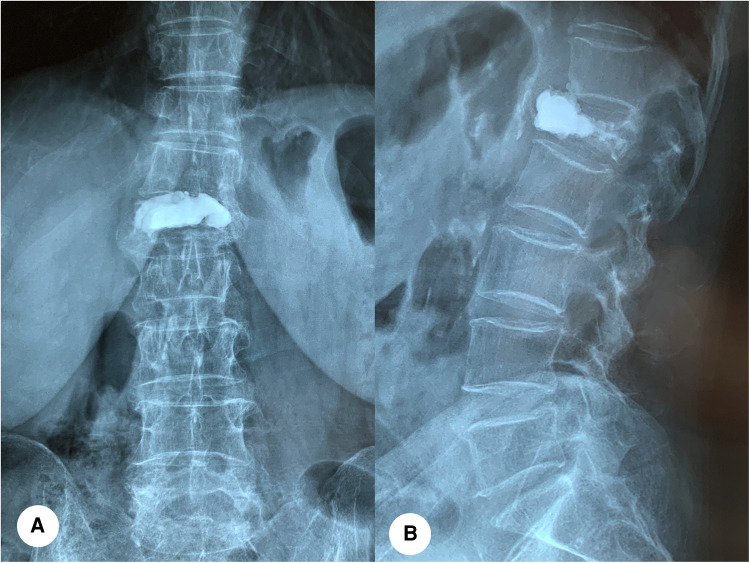
A 79-year-old female with bone cement displacement. (**A,B**): Anteroposterior and lateral x-ray radiographs showed that anterior displacement of bone cement of the L1 vertebra.

A total of 1,538 patients with OVCF were included, including 377 males and 1,161 females, aged 45–115 years (mean = 71 years). Based on the imaging findings of patients with new back pain, they were divided into the bone cement displaced group (*n* = 78) and bone cement non-displacement group (*n* = 1460). A typical case of cement displacement is shown in Figure 1. All patients signed the informed consent form. This study was approved by the Medical Ethics Committee of Honghui Hospital affiliated to Xi’an Jiaotong University (No.2021086).

### Operation method

The patient was placed in a prone position, and the responsible vertebra was located through fluoroscopy combined with preoperative imaging examination. The position and angle of needle insertion were determined and marked on the skin. Routine disinfection towels were used for local anesthesia with 10 g/L lidocaine hydrochloride. The needle was inserted at the projection location of the body surface of unilateral or bilateral pedicle of the affected vertebra. Lateral fluoroscopy confirmed that the needle exceeded the posterior edge of the vertebra, and orthotopic fluoroscopy confirmed that the tip did not pass through the medial cortex of the pedicle, and continued the needle insertion. Lateral fluoroscopy confirmed the satisfactory position of the needle, pull out the needle core. Surgical procedures for patients with PVP: under the fluoroscopy of the C-arm x-ray machine, the suitable bone cement material was injected into the responsible vertebra with a pressure syringe. Surgical procedures for patients with PKP: A percutaneous cannula is inserted into the vertebral body *via* the pedicle *via* a balloon dilated percutaneous vertebroplasty package. The balloon was inserted through the working cannula. After the correct position of the balloon was determined under fluoroscopy, the contrast agent was injected and the balloon was expanded to reset the compressed vertebral body. After the balloon expansion was completed under fluoroscopy, the pressure was stopped, the balloon was withdrawn, and appropriate bone cement was injected through the cannula to fill the gap. The injection device was removed after the bone cement distribution was observed satisfactorily under C-arm x-ray machine fluoroscopy. The wound was compressed to stop bleeding, and a sterile dressing was applied. All cases received regular anti-osteoporosis treatment (calcium + vitamin D + diphosphate) after the operation.

### Evaluation index

Univariate analysis was used to analyze gender, age, BMI, underlying illness, diseased segment, number of fractured vertebrae, surgical methods and approach, recovery rate of vertebral height (anterior height of fractured vertebra/([upper adjacent vertebral anterior height + lower adjacent vertebral anterior height]/2)), preoperative and postoperative Cobb angle, restoration of Cobb angle ((preoperative Cobb angle−postoperative Cobb angle)/ preoperative Cobb angle), cement leakage, bone cement viscosity, bone cement diffuse ratio (bone cement dispersion volume/vertebral volume), bone cement interweaving (trabecular volume/bone cement mass volume), sagittal position of cement filling, targeted position of cement, the distance between bone cement and upper and lower lamina, bracing time, preoperative and postoperative BMD, restoration of BMD ((preoperative BMD−postoperative BMD)/ preoperative BMD) and postoperative osteoporosis treatment with the displacement of bone cement after PVA. The quantitative assignment of the above-related factors is shown in Table 1. Multivariate logistic regression analysis was used to determine the independent risk factors of bone cement displacement after PVA.

**Table 1:** Univariate analysis of postoperative bone cement displacement.

Variables	Displacement Group (*n* = 78)	Non-displacement Group (*n* = 1,460)	*χ*^2^/*t* value	*P*-value
Gender			0.453	0.504
Male	25	352		
Female	53	1,108		
Age (years)	69.8 ± 6.8	68.9 ± 7.9	0.897	0.380
BMI (kg/m^2^²)	22.26 ± 1.97	22.72 ± 1.22	2.303	0.022
Underlying illness			4.642	0.200
Hypertension	18	247		
Diabetes	9	113		
Heart disease	14	244		
Others	37	856		
Diseased segment			6.090	0.048
Thoracic	21	242		
Thoracolumbar	46	1,028		
Lumbar spine	11	190		
Number of fractured vertebrae	2.10 ± 0.61	1.28 ± 0.59	11.939	<0.001
Surgical methods			9.480	0.002
PKP	71	1,108		
PVP	7	352		
Surgical approach			0.700	0.401
Unilateral	47	809		
Bilateral	31	651		
Recovery rate of vertebral height (%)	56.89 ± 8.10	49.04 ± 5.25	8.465	<0.001
Preoperative Cobb angle (°)	22.56 ± 4.23	22.39 ± 3.96	0.368	0.713
Postoperative Cobb angle (°)	13.31 ± 3.22	15.23 ± 3.84	5.077	<0.001
Restoration of Cobb angle (%)	40.59 ± 8.56	30.28 ± 11.33	8.200	<0.001
Cement leakage			14.942	0.001
Anterior leakage	29	285		
Lateral leakage	5	98		
Posterior leakage	6	105		
Non-leakage	38	972		
Bone cement viscosity			14.660	<0.001
High viscosity	31	898		
Low viscosity	47	562		
Bone cement diffuse ratio	0.21 ± 0.04	0.44 ± 0.09	28.736	<0.001
Interweaving degree of bone cement	0.16 ± 0.05	0.38 ± 0.07	28.789	<0.001
Sagittal position of cement filling			7.920	<0.001
Anterior 1/3	33	321		
Anterior middle 2/3	37	868		
Whole vertebral body	8	271		
Targeted position of cement			9.08	0.010
Targeted injection of upper and lower endplates	5	201		
Non-targeted injection	52	726		
Combination of both	21	533		
The distance between bone cement and upper and lower lamina	0.32 ± 0.08	0.19 ± 0.04	13.654	<0.001
Bracing time (days)	9.8 ± 3.1	13.6 ± 2.5	9.596	<0.001
Preoperative BMD (T score)	−3.94 ± 0.78	−3.53 ± 0.34	5.965	<0.001
Postoperative BMD (T score)	−2.95 ± 0.53	−2.03 ± 0.24	15.247	<0.001
Restoration of BMD (%)	32.74 ± 8.33	31.22 ± 6.56	1.686	0.117
Postoperative osteoporosis treatment			29.093	<0.001
Yes	22	864		
No	56	596		

**Figure 2:**
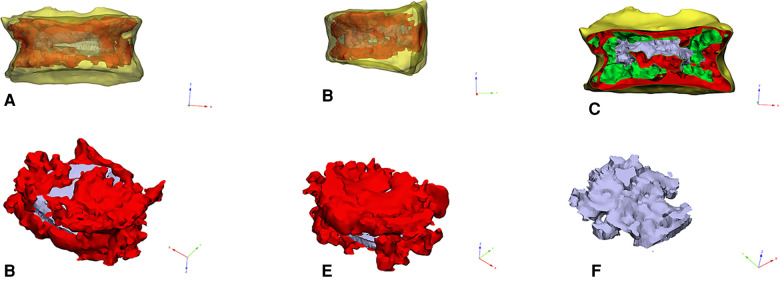
(**A–C**): The picture of the three-dimensional geometric model of the responsible vertebral body. (**D,E**): The picture of intravertebral bone cement model. (**F**): The picture of trabecular bone model in bone cement.

The diffusion ratio of bone cement was calculated using the method of Wang (12), that is, diffusion ratio of bone cement = bone cement dispersion volume/vertebral volume. Calculation method of interweaving degree of bone cement: by collecting preoperative and postoperative CT data in DICOM format, the responsible vertebral body model (A, B) and the three-dimensional model of bone cement mass (C) were reconstructed by Mimics software (Materialise Company, Belgium). The reconstructed model was imported into Geomagic Studio 12 software (Raindrop, USA) for processing. Step format files were imported into 3d modeling software Solidworks 2016 (Dassault, France) for reconstruction of cortical bone, cancellous bone, and other anatomical structures. Finally, the finite element analysis software ANSYS (ANSYS software Company, American) was used to conduct mesh division and assign value of bone cement properties to construct the bone trabecular model wrapped in bone cement mass (D). The trabecular volume and bone cement mass volume were calculated respectively (accurate to 0.01 ml). That is, the bone cement interweaving is defined as trabecular volume/bone cement mass volume. The three dimensional finite element model is shown in Figure 2.

### Statistical analysis

Continuous data are expressed as means ± standard deviations, and the independent samples *t*-test was used for comparison between groups. Categorical data were analyzed by a chi-square test. A univariate analysis was used to identify potential influencing factors for bone cement displacement after PVA. A multivariate logistic regression analysis was conducted using the variables with statistical significance in the univariate analysis. The accuracy was assessed by receiver operating curve (ROC) by plotting sensitivity against 1- specificity. All statistical analyses were performed using SPSS Statistics for Windows, version 26.0 (IBM Corp, Armonk, NY, USA). All tests were bilateral. A *P* value of <0.05 was considered statistically significant.

## Results

### Univariate analysis

There was no significant difference in sex and age between the displacement and non-displacement groups (*P* > 0.05), as well as in underlying disease, surgical approach, preoperative Cobb angle, restoration of BMD (all *P* > 0.05). BMI, diseased segment, number of fractured vertebrae, surgical method, recovery rate of vertebral height, postoperative Cobb angle, restoration of Cobb angle, cement leakage, bone cement viscosity, bone cement diffuse ratio, degree of bone cement interweaving, sagittal bone cement placement, targeted location of bone cement, the distance between the bone cement and the upper and lower endplates, the time of wearing brace, preoperative and postoperative BMD and postoperative osteoporosis treatment were correlated with bone cement displacement after PVA (*P* < 0.05).

### Multivariate analysis

**Figure 3:**
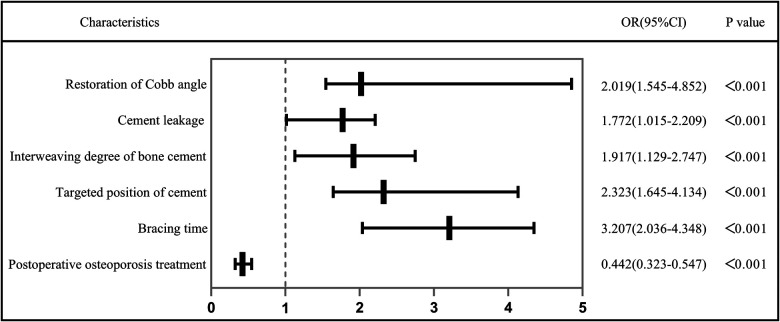
Risk factors of postoperative bone cement mass displacement.

The independent risk factors that positively correlated with bone cement displacement development after PVA were as follows: High restoration of Cobb angle (OR = 2.019, 95%[CI] 1.545–4.852, *P* < 0.001), cement leakage (anterior edge) (OR = 1.727, 95%[CI] 1.05–2.20, *P* < 0.001), small degree of bone cement interweaving (OR = 1.917, 95%[CI] 1.129–2.747, *P* < 0.001), non-targeted location of bone cement (OR = 2.323, 95%[CI] 1.645–4.134, *P* < 0.001), short duration of brace wearing (OR = 3.207, 95%[CI] 2.036–4.348, *P* < 0.001) and postoperative osteoporosis treatment (OR = 0.422, 95% CI = 0.323–0.547, *P* < 0.001). Table 2 and Figure 3 show the results of multivariate analysis.

**Table 2:** Multivariate logistic regression analysis of postoperative bone cement displacement.

Variables	OR	*P*-value	CI 95% l
Surgical methods	1.562	0.104	0.857–2.440
Diseased segment	1.892	0.057	0.884–4.973
Number of fractured vertebrae	1.752	0.055	0.544–2.783
Recovery rate of vertebral height	1.878	0.072	0.642–4.072
Postoperative Cobb angle (°)	1.023	0.066	0.673–1.237
Restoration of Cobb angle (%)	2.019	<0.001	1.545–4.852
Cement leakage	1.772	<0.001	1.015–2.209
Bone cement diffuse ratio	1.846	0.091	0.592–3.391
Bone cement viscosity	1.227	0.105	0.929–2.147
Interweaving degree of bone cement	1.917	<0.001	1.129–2.747
Sagittal position of cement filling	1.897	0.094	1.056–2.471
Targeted position of cement	2.323	<0.001	1.645–4.134
The distance between bone cement and upper and lower lamina	1.788	0.241	0.838–3.962
Bracing time (days)	3.207	<0.001	2.036–4.348
Preoperative BMD	2.456	0.069	1.807–3.503
Postoperative BMD	1.838	0.066	0.239–2.629
Postoperative osteoporosis treatment	0.422	<0.001	0.323–0.547

### Predictive performance

**Figure 4:**
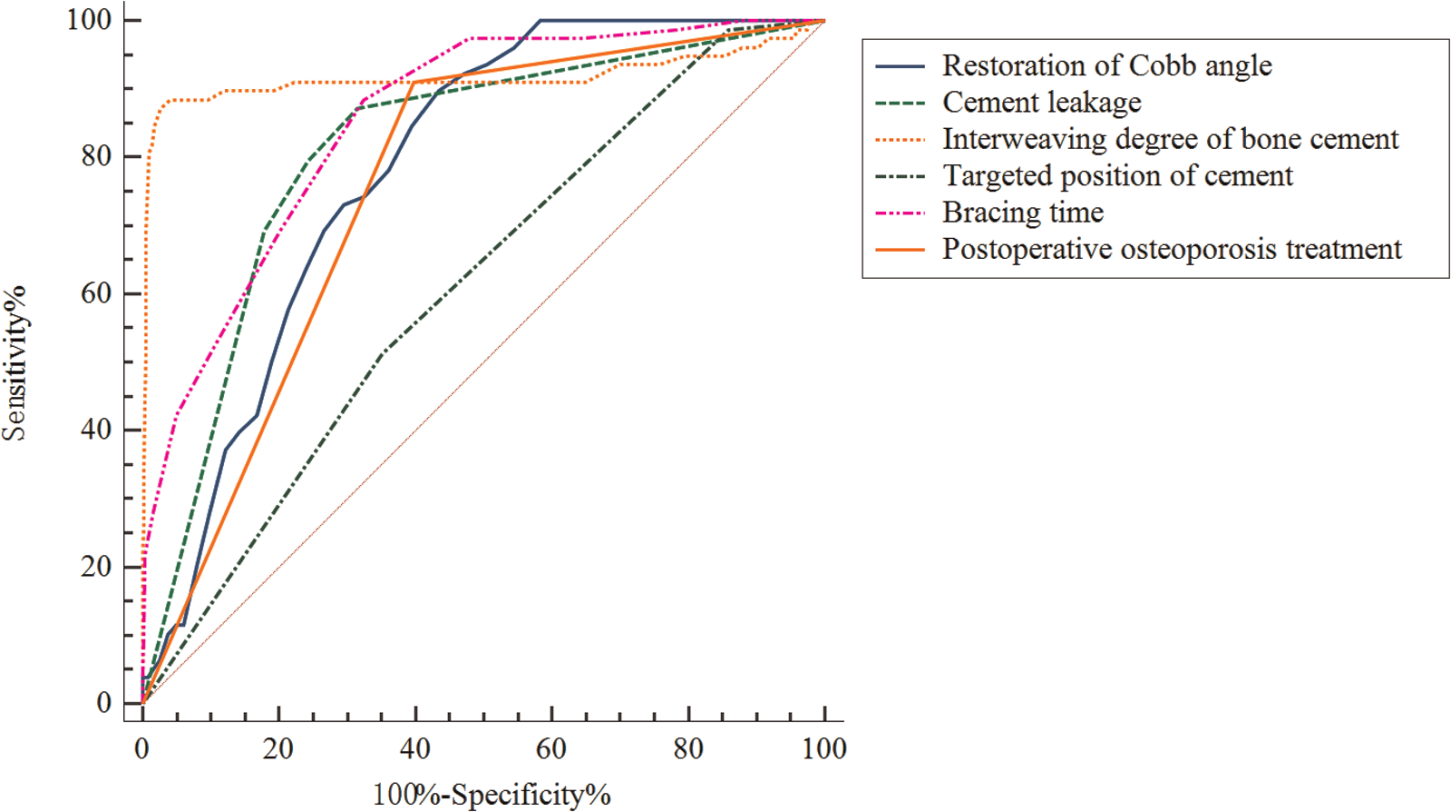
Receiver operating curves for the prediction performance of the multiple logistic regression model.

ROC curve analysis was used to analyze the predictive performance of high restoration of Cobb angle, cement leakage (anterior edge), small degree of bone cement interweaving, non-targeted location of bone cement, short duration of brace wearing and non-postoperative osteoporosis treatment (Figure 4). The optimal cutoffs and corresponding sensitivity and specificity and AUC were listed in Table 3. The optimal cut-off value of restoration of Cobb angle for predicting cement displacement was 32.5, which yielded sensitivity and specificity of 89.7% and 56.5%, respectively. The sensitivity and specificity of cement leakage (anterior edge) to predict cement displacement were 87.2% and 68.5%, respectively. The optimal cut-off value of interweaving degree of bone cement for predicting cement displacement was 0.245, which yielded sensitivity and specificity of 88.5% and 96.0%, respectively. The sensitivity and specificity of non-targeted location of bone cement to predict cement displacement were 51.3% and 64.8%, respectively. The optimal cut-off value of short duration of brace wearing for predicting cement displacement was 12.5, which yielded sensitivity and specificity of 88.5% and 67.5%, respectively. The sensitivity and specificity of non-postoperative osteoporosis treatment to predict cement displacement were 91.0% and 60.2%, respectively. These results demonstrated that the combined index could predict the bone cement displacement significantly.

**Table 3:** Predictive performance of related indexes on bone cement displacement.

Variables	AUC	Cut-off	*95%*CI	Sensitivity	Specificity	*P-*value
Restoration of Cobb angle	0.784	32.5	0.747–0.821	0.897	0.565	<0.001
Cement leakage	0.811	–	0.764–0.859	0.872	0.685	<0.001
Interweaving degree of bone cement	0.917	0.245	0.864–0.970	0.885	0.960	<0.001
Targeted position of cement	0.610	–	0.552–0.669	0.513	0.648	0.001
Bracing time	0.854	12.5	0.816–0.892	0.885	0.675	<0.001
Postoperative osteoporosis treatment	0.756	–	0.712–0.800	0.910	0.602	<0.001

## Discussion

As a surgical treatment for OVCF, PVA is easy to perform and has a good clinical effect. As a rare complication of PVA, bone cement displacement development is a troublesome concern for patients and clinicians. To date, the influencing factors of bone cement displacement have not been studied at home and abroad, only cases of bone cement displacement after PVA have been reported. Therefore, in combination with clinical practice, we took patient characteristics, fracture characteristics and treatment variables as entry points to thoroughly study the influencing factors of bone cement displacement after PVA. In our study, the independent risk factors for bone cement displacement after PVA by including factors, such as high restoration of Cobb angle, cement leakage (anterior edge), small degree of bone cement interweaving, non-targeted location of bone cement, short duration of brace wearing and non-postoperative osteoporosis treatment.

### Restoration of Cobb angle

Cobb angle is one of the most frequently used factors reflecting compressive and kyphotic deformity caused by a vertebral fracture. Kang et al. (13) found that there were 20 patients of vertebral refracture with excessive Cobb angle restoration in 60 patients after PVA and concluded that high restoration of Cobb angle was the risk factor. High restoration of Cobb angle might be due to that a smaller Cobb angle postoperatively would result in numbness in vertebral internal structure and imbalanced stress, which lead to imbalance stress of sagittal spine and increasing the risk of cement displacement. In our study, we found that high restoration of Cobb angle (OR = 2.019, 95%[CI] 1.545–4.852, *P* < 0.001) was a risk factor for cement displacement after PVA. Cao et al. (14) also found that the small improvement rate of Cobb angle after operation would lead to the change of spinal stress and the global sagittal balance. Therefore, we recommend that physicians focus on local kyphosis correction to restore the balance of global sagittal balance, rather than solely pursuing vertebral height recovery and kyphosis correction of fractured vertebrae.

### Cement leakage

To date, the research on bone cement leakage after vertebral body augmentation is still controversial. Zhang et al. (15) summarized the current research progress of bone cement leakage from different perspectives, such as classification, influencing factors, and prevention and control measures. We focused on the anterior leakage of bone cement and analyzed its correlation with bone cement displacement. The results of this study showed that the anterior leakage of bone cement (OR = 1.727, 95%[CI] 1.015–2.209, *P* < 0.001) was significantly correlated with the occurrence of bone cement displacement after PVA. Previous studies have shown that vertebral cortical bone defect is an independent risk factor for bone cement leakage (16). If there is a defect in the bone cortex of the anterior edge of the vertebral body, the bone cement leakage may further develop into bone cement displacement. Wang et al. (9) believed that the defect of anterior cortex may increase the probability of cement displacement under weight-bearing conditions. According to Denis’s three-column theory, the anterior margin of the vertebral body is an important part of the anterior column of the spine, and the anterior 1/3 of the vertebral body is the area that bears the highest pressure (17). We also indirectly confirm this result by establishing a three-dimensional finite element model. When the leakage of the leading edge of bone cement occurs, it affects the biomechanics of the spine to a certain extent, and the risk of cement displacement increases over time. Therefore, it was suggested to carefully examine the imaging data of the patients before operation, including x-ray, CT and MRI to determine the integrity of the vertebral wall, the severity of collapse and whether there is a more bony fragments. If the bone cortical defect is found, special attention should be paid to avoid the leakage of the leading edge of bone cement during operation, which can reduce the risk of cement displacement to a certain extent.

### Interweaving degree of bone cement

At present, there are different methods to evaluate the dispersion distribution of bone cement after PVA. More and more scholars believe that the diffusion of bone cement plays an important role in surgical efficacy and postoperative complications (18, 19). Wang et al. (12) put forward the concept of bone cement diffusion ratio, which is considered to reflect the dispersion distribution of bone cement in the vertebral body more objectively and accurately. There is no doubt about the application of the concept of bone cement diffusion ratio in fresh OVCF because the bone cement diffusion ratio is a relatively stable value in this kind of fracture. However, when the displacement of bone cement mass occurs, the concept of bone cement dispersion ratio is no longer applicable. Clumps of bone cement are distributed in the cracks. Although the diffusion of bone cement is relatively high, on the contrary, the anchoring strength of bone cement in the vertebral body is relatively low, and the actual solid anchoring strength of bone cement with trabecular bone is very small. At this time, the interweaving degree of bone cement can well reflect the anchoring degree and stability of bone cement and bone trabecula. The interweaving degree of bone cement is measured by three-dimensional finite element method, which was reconstructed by digital orthopaedic technique. The trabecular volume and the volume of bone cement mass wrapped in bone cement mass after PVA were calculated through the three-dimensional finite element, which were used as an objective index to evaluate the close degree of cross-coupling between bone cement and bone trabecula. When the ratio is larger, it means that the bone cement in the vertebral body is anchored more closely with the bone trabecula. The more firm the mass is, the less likely it is to shift the bone cement mass. On the contrary, when the ratio is smaller, it means that the bone cement in the vertebral body is loosely anchored with trabecular bone, and the displacement of bone cement mass is more likely to occur. The results of this study showed that the interweaving degree of bone cement <0.2 (OR = 1.917, 95%[CI] 1.129–2.747, *P* < 0.001) was significantly related to the bone cement displacement after PVA. Therefore, the interweaving degree of bone cement <0.2 is an independent risk factor for postoperative loosening and displacement of bone cement. Accordingly, the bone cement is dispersed evenly and anchored with the bone trabecula as far as possible during the surgery to avoid the formation of bone cement mass may reduce the occurrence of bone cement displacement.

### Targeted position of cement

The results also showed that non-targeted injection of bone cement (OR = 2.323, 95%[CI] 1.645–4.134, *P* < 0.001) was an independent risk factor for postoperative displacement of bone cement. Biomechanical studies have shown that the bone mineral density of the fracture compression area is significantly increased, the strength of the targeted injection of bone cement is more similar to that of the fracture in the compression area, the anchoring force is stronger, and the interlacing degree of the targeted injection area is also higher (20), which also indicates that the risk of non-targeted injection of bone cement displacement may be greater. Yu et al. (21) also believed that target puncture technique could make the bone cement to diffuse through trabecular space to the non-fracture area and the endplate bone tissue and to blend with the surrounding cancellous bones more densely to improve the efficacy and safety of surgery. Therefore, targeted injection of bone cement is recommended to strengthen the anchoring effect of bone cement and trabecula of injured vertebrae.

### Bracing time

Patient dependence has always been the main factor affecting the prognosis of patients. Zhang et al. (22) believed that thoracolumbar bracing could not improve the prognosis of patients in terms of quality of life and postoperative complications. However, the results of this study showed that wearing the brace for a long time (OR = 3.207, 95%[CI] 2.036–4.348, *P* < 0.001) as required after surgery can significantly reduce the occurrence of bone cement displacement. To investigate the reason, we believe that thoracolumbar brace can reduce trunk movement, improve bone alignment, and reduce vertebral bone tissue pressure, trabecular friction, and facet joint movement to a certain extent. In addition, when the patients were bending and bearing weight after operation, the interfacial stress between bone cement and trabeculae increased due to the flexion of injured vertebrae, which increased the risk of cement displacement (23). Over time, the relative stability between bone cement and trabeculae may be broken through. Its contact surface hardens, that is, new ossification cladding and hardened tension bands are formed around the bone cement mass, which adjusts and limits the absolute displacement of the bone cement and forms a relative displacement that can be detected by imaging examination. Of course, when the ossification envelope is re-fractured, the obvious displacement of bone cement after breaking through the limit may be the potential reason for the significant displacement of some bone cement in this study.

### Postoperative osteoporosis treatment

Osteoporosis can be caused by a variety of factors, the main cause of which is bone loss. Once this happens, it is difficult to prevent or reverse progression by patient’s regulatory mechanisms solely. However, anti-osteoporosis drugs can effectively prevent or even reverse bone loss (1). Hoff et al. (24) recruited 28,461 volunteers and analyzed the effect of anti-osteoporosis medications on the incidence of fractures, showing that fractures incidence was significantly higher in the untreated group than in the treated group, thus highlighting the necessity and importance of anti-osteoporosis drug therapy for bone loss. Hsu et al. (25) showed that lower BMD was associated with higher mortality risk in patients with poor adherence. In our study, postoperative osteoporosis treatment (OR = 0.422, 95% CI = 0.323–0.547, *P* < 0.001) was a protective factor for cement displacemen. A large number of studies have shown that low BMD is not only a significant risk factor for complications after spinal surgery, but also related to patient satisfaction (26). In addition, standardized anti-osteoporosis treatment after operation plays an important role in improving the prognosis and survival rate of patients (27). Therefore, in order to prevent cement displacement after PVA, clinicians should not only recommend personalized anti-osteoporosis treatment according to the specific situation of patients, but also emphasize patient compliance.

For this rarely seen disease, treatment is challenging and there is no consolidate method in clinical practice. Our main treatment options are as follows: for bone cement displacement without neurological damage, conservative management strategies such as bed rest, narcotic analgesics, and use of a brace are often used as initial treatments. For patients with symptoms of nerve damage, patients often need to undergo open posterior, anterior, or even anterior and posterior revision surgery to remove the displaced bone cement, reconstruct spinal stability, and restore the spinal sequence and fusion. Furthermore, bone cement screw system combined with vertebroplasty we designed was also used in these patients with neurological damage (28).

### Limitations

This study is a retrospective single-center study with a small number of cases and only included the cases with anterior displacement of bone cement, excluding lateral or posterior displacement. Therefore, a multi-center, large-sample study is needed to provide more convincing data. The measurement of the interweaving degree of bone cement proposed by us requires preoperative and postoperative CT examination, which increases a certain amount of radiation to the patients. It needs to be improved to avoid radiation damage to the patients in the future.

## Conclusion

High restoration of Cobb angle, cement leakage (anterior edge), small degree of bone cement interweaving, non-targeted location of bone cement, short duration of brace wearing and non-postoperative osteoporosis treatment were the independent risk factors of bone cement displacement after PVA. In the clinic, the corresponding intervention measures are implemented to reduce the risk of bone cement displacement after PVA, so as to improve the prognosis and quality of life of the patients.

## Data Availability

The raw data supporting the conclusions of this article will be made available by the authors, without undue reservation.
